# Targeting urine output and 30-day mortality in goal-directed therapy: a systematic review with meta-analysis and meta-regression

**DOI:** 10.1186/s12871-017-0316-4

**Published:** 2017-02-10

**Authors:** Esther N. van der Zee, Mohamud Egal, Diederik Gommers, A. B. Johan Groeneveld

**Affiliations:** 1 Department of Intensive Care, Erasmus MC, University Medical Center Rotterdam, Rotterdam, The Netherlands; 2 Erasmus MC, Room H-602, P.O. Box 2040, 3000 CA Rotterdam, The Netherlands

**Keywords:** Oliguria, Mortality, Perioperative care, Critical care

## Abstract

**Background:**

Oliguria is associated with a decreased kidney- and organ perfusion, leading to organ damage and increased mortality. While the effects of correcting oliguria on renal outcome have been investigated frequently, whether urine output is a modifiable risk factor for mortality or simply an epiphenomenon remains unclear. We investigated whether targeting urine output, defined as achieving and maintaining urine output above a predefined threshold, in hemodynamic management protocols affects 30-day mortality in perioperative and critical care.

**Methods:**

We performed a systematic review with a random-effects meta-analyses and meta-regression based on search strategy through MEDLINE, EMBASE and references in relevant articles. We included studies comparing conventional fluid management with goal-directed therapy and reporting whether urine output was used as target or not, and reporting 30-day mortality data in perioperative and critical care.

**Results:**

We found 36 studies in which goal-directed therapy reduced 30-day mortality (OR 0.825; 95% CI 0.684-0.995; *P* = 0.045). Targeting urine output within goal-directed therapy increased 30-day mortality (OR 2.66; 95% CI 1.06-6.67; *P* = 0.037), but not in conventional fluid management (OR 1.77; 95% CI 0.59-5.34; *P* = 0.305). After adjusting for operative setting, hemodynamic monitoring device, underlying etiology, use of vasoactive medication and year of publication, we found insufficient evidence to associate targeting urine output with a change in 30-day mortality (goal-directed therapy: OR 1.17; 95% CI 0.54-2.56; *P* = 0.685; conventional fluid management: OR 0.74; 95% CI 0.39-1.38; *P* = 0.334).

**Conclusions:**

The principal finding of this meta-analysis is that after adjusting for confounders, there is insufficient evidence to associate targeting urine output with an effect on 30-day mortality. The paucity of direct data illustrates the need for further research on whether permissive oliguria should be a key component of fluid management protocols.

**Electronic supplementary material:**

The online version of this article (doi:10.1186/s12871-017-0316-4) contains supplementary material, which is available to authorized users.

## Background

Textbooks and guidelines frequently recommend urine output as a parameter to guide fluid administration, since decreased organ perfusion may decrease urine output in an attempt to maintain intravascular volume [1–3]. However, a suboptimal hemodynamic status is not always the cause of oliguria. In recent years, the concept of an association between intraoperative urine output and postoperative acute kidney injury has been challenged [4–6]. As a result, advocacy for permissive oliguria has increased, for example to include permissive oliguria in the early recovery after surgery (ERAS) protocols [7–9].

Our group has previously published meta-analyses concerning the effects of targeting urine output on acute renal failure or acute kidney injury [10, 11]. A frequent remark on these meta-analyses was that while targeting urine output may not have an effect on preventing acute kidney injury, there is increasing evidence that reduced urine output is a risk factor for mortality [12–15]. Especially in critically ill patients, the occurrence and severity of oliguria is associated with an increase in mortality. Whether the association between urine output and outcome is due to a causal relation or rather an epiphenomenon is yet to be determined. Nevertheless, fluids and vasoactive medication are often administered to patients with a decrease in urine output to guarantee and maintain adequate perfusion. However, whether urine output is a useful target for fluid management remains doubtful, especially when direct measures related to cardiac output and oxygen delivery are available.

We hypothesize that including urine output as a target does not decrease 30-day mortality in perioperative and critical care. This study aims to investigate whether including urine output as a target in fluid management protocols reduces 30-day mortality in perioperative and critical care.

## Methods

### Search strategy

We conducted a systematic literature search of MEDLINE by using PubMed (1966 – present) and EMBASE (1980 – present). There were no studies directly investigating the effect on 30-day mortality by urine output as fluid management target in a perioperative or critical care protocol. Therefore, to determine the effect of urine output as a target, all studies comparing goal-directed therapy (GDT) and conventional fluid management (CFM) and reporting within 30-day mortality were identified. The last search was performed in May 2016. No limits for publication date or language were used. Additional file 1: Table S1 and Table S2, shows the strategy for the MEDLINE and EMBASE database. The ‘related articles’ function in PubMed provided us with the opportunity to identify eligible studies that were not found by the main search queries. All references of the identified articles and review articles were hand searched to avoid missing relevant trials. We screened the title and abstract of the studies found in the databases to determine whether GDT was compared to CFM and to establish whether mortality was reported. We used the full text of the article in case of uncertainty about the therapy or mortality.

### Study selection

The search was performed by two authors (E.Z., M.E.). Disagreements were resolved by consensus or if necessary by a third author (ABJG). We included randomized controlled trials during perioperative or critical care into our main analysis, whereas observational studies have been collected and are reported in Additional file 1. Animal studies, pediatric trials (<18 years), articles written in another language than English, studies unavailable as full-text, and studies in which mortality data was not clearly described were excluded. Due to the difficulty of using urine output as a parameter after administration of diuretics, the use of diuretic drugs to increase urine output was not allowed during the intervention period. Therefore, studies using diuretics during the intervention period were excluded. Although a full description of the protocol was not required, the hemodynamic targets in the CFM arm had to be clearly reported. We excluded studies which described the CFM arm as ‘standard treatment’ without further elaboration. Quality assessment was performed using the Cochrane Collaboration’s tool for assessing risk of bias [16].

### Definitions

Goal-directed therapy was defined as any hemodynamic optimization strategy in the perioperative and critical care setting, utilizing parameters related to cardiac output and oxygen delivery, either exclusively or in combination with classical parameters such as blood pressure and heart rate, irrespective of the device or method used to measure these parameters. Urine output as a target was defined as achieving and maintaining urine output using fluids and vasoactive medication above a predefined threshold. We did not redefine the urine output thresholds and used the thresholds as set by the respective studies. No distinction was made between isolated oliguria or urine output in combination with other hemodynamic parameters. We defined mortality as death by all causes within 30 days after inclusion. In case mortality was reported as ‘intensive care mortality’ or ‘in-hospital mortality’, we used the respective length of stay data to determine the survival duration. Studies in which more than 75% of the patients were admitted for less than 30 days were considered for reporting 30-day mortality.

### Data collection

Two authors (E.Z., M.E.) extracted the following variables: total study population, size of GDT arm, size of CFM arm, type of patients, timing of the intervention period, definition of GDT and CFM, urine output target criteria, total amount of fluids infused per study arm, intraoperative and postoperative urine output data, treatment targets in both study arms, definition of mortality and number of deaths.

### Data synthesis

All selected studies were divided into three groups for the main forest plot based on whether oliguria reversal was included as a target in a study protocol: trials comparing GDT and CFM in which both the GDT protocol and the CFM protocol did not include urine output as a target, articles in which urine output was only targeted in the CFM protocol, and articles in which GDT and CFM treatment arms both included urine output as target. We analyzed whether there was a difference in 30-day mortality between the two treatment arms and in the targeting urine output subsets. A funnel plot was conducted to identify asymmetry. If publication bias was detected, possible missing studies were identified by using the ‘trim and fill’ method.

To investigate the effect of targeting urine output in CFM and in GDT on mortality, a meta-regression model was performed to estimate a regression equation with 30-day mortality as outcome and the use of urine output as a target as a variable for GDT and CFM. This meta-regression model was then adjusted with study setting, hemodynamic monitoring device used, underlying etiology, use of vasoactive medication and year of publication as covariates in the regression equation. The year of publication variable was centered on the mean year of publication, which was 2008.

Due to the various threshold values used as the urine output target, we performed a sensitivity analysis excluding studies utilizing a urine output target different from the conventional standard of 0.5 ml/kg/h. This sensitivity analysis was performed for both the meta-analysis as well as the meta-regression analysis.

### Statistical analysis

For each study odds ratios (OR) and 95% confidence intervals (CI) were calculated, based on their sample sizes of the GDT and CFM and the reported mortality in those treatment arms. All meta-analyses were conducted as random effect meta-analyses in R (version 3.2.1) using the metafor package [17, 18]. The Sidik-Jonkman estimator was used in combination with Knapp & Hartung adjustment to improve estimates of the heterogeneity variance due to the low number of studies included [19, 20]. In studies with a count of zero in one of the treatment arms, 0.5 was added to all frequencies. Heterogeneity between the trials was analyzed using the I^2^ statistic and interpreted using thresholds as defined in the Cochrane Handbook [21]. A trial sequence analysis was performed to account for random error. Optimal sample size – i.e., information size – was determined using alpha = 0.05 and power of 0.80 for a relative risk reduction of 25%. Due to the Knapp-Hartung adjustment utilizing a t-distribution, we converted the t-value to a z-score using a nominal p-value approach for the trial sequence analysis. Quality of evidence was assessed using the GRADE system [22]. We used a random-effects meta-regression model with targeting urine output, study setting, hemodynamic monitoring device used, underlying etiology, use of vasoactive medication and year of publication as covariates and fluid management protocol (GDT or CFM) as the inner grouping variable and study as the outer grouping variable to test the effect of the moderators on 30-day, using a bivariate approach which has been described earlier [23]. This method resulted in separate regression equations for the 30-day mortality risk in GDT and in CFM. For the sensitivity analysis for studies with a urine output target of 0.5 ml/kg/h, we repeated the meta-analysis and meta-regression analysis. Odds ratios were considered statistically significant when their 95% CI did not include 1.00 and the corresponding *P*-value was less than 0.05.

## Results

**Fig. 1:**
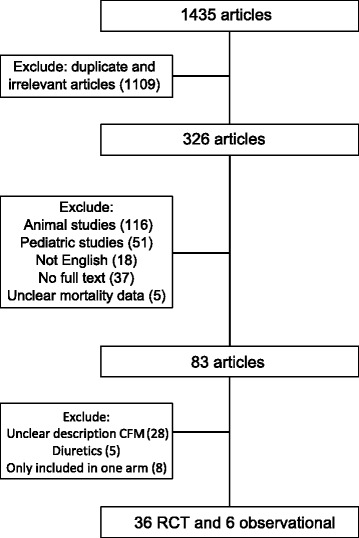
Flow chart of study selection. CFM: conventional fluid management; RCT: randomized controlled trial

**Fig. 2:**
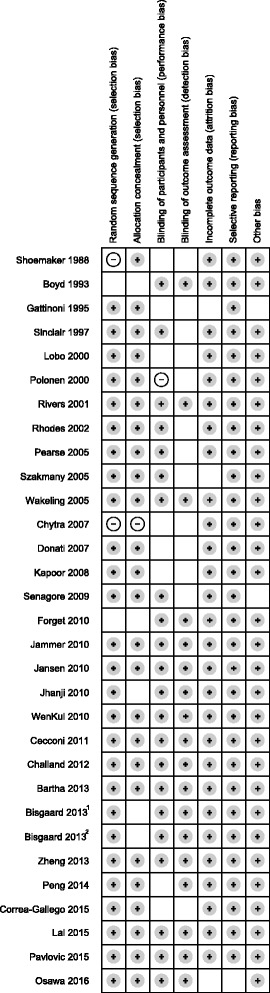
Risk of bias assessment. Risk of bias assessment performed with the Cochrane Collaboration tool [16]. Because there are two studies by Bisgaard et al. published in 2013, ^1^ marks reference [35], and ^2^ marks reference [56]. Gray circle: low risk of bias; blank: unclear risk of bias; white circle: high risk of bias

Our search strategy resulted in 1435 articles. A total of 326 remained after excluding duplicates and irrelevant articles. After removing studies which met our exclusion criteria, 83 articles remained. An additional 41 studies were excluded based on the usage of diuretics, or the absence of a description of the hemodynamic parameters in the CFM arm (Fig. 1). Table 1 shows the characteristics of the remaining 36 randomized controlled trials. Thirteen studies [24–36] did not target urine output in either GDT or CFM; seven studies [37–43] only targeted urine output in the CFM protocol; and sixteen studies [44–59] targeted urine output in both protocols. Hemodynamic monitoring devices and parameters used in the included studies are reported in Table 2. The amount of fluids infused during GDT and CFM in each study is reported in Additional file 1: Table S3. The risk of bias assessment is shown in Fig. 2. Of the 23 studies which included urine output as a target, fifteen studies had a threshold of 0.5 ml/kg/h (Table 2). The data of the limited studies in which the amount of urine output was reported are collected in Additional file 1: Table S4. The data on the six observational studies [60–65] are reported in Additional file 1: Table S5 and Table S6.

**Table 1:** Characteristics of studies included

Study	Total number	Type of patient	Timing	Mortality follow up
Not targeting urine output in either protocol				
Sinclair 1997 [24]	40	Orthopedic	intra	30 days
Polonen 2000 [25]	393	Cardiac	post	28 days
Rhodes 2002 [26]	201	Critically ill	ICU	28 days
Pearse 2005 [27]	122	High risk	post	28 days
Szakmany 2005 [28]	40	Abdominal	Intra	3 days postoperative
Wakeling 2005 [29]	128	Abdominal	intra	30 days
Forget 2010 [30]	86	Abdominal	intra	30 days
WenKui 2010 [31]	214	Abdominal	intra, post	30 days
Cecconi 2011 [32]	40	Orthopedic	intra	28 days
Challand 2012 [33]	236	Abdominal	Intra	30 days
Bartha 2013 [34]	149	Orthopedic	intra	30 days
Bisgaard 2013 [35]	70	Abdominal	intra, post	30 days
Lai 2015 [36]	221	Abdominal	Intra	30 days
Targeting urine output only in CFM				
Bishop 1995 [37]	115	Trauma	post, ICU	in-hospital (95% < 15 days)
McKendry 2004 [38]	174	Cardiac	post	30 days
Benes 2010 [39]	120	High Risk	intra	30 days
Mayer 2010 [40]	60	High Risk	Intra	in-hospital (95% < 30 days)
McKenny 2013 [41]	101	Abdominal	Intra	30 days
Zakhaleva 2013 [42]	74	Abdominal	Intra	30 days
Osawa 2016 [43]	126	Cardiac	Intra, post	30 days
Targeting urine output in both protocols				
Shoemaker 1988 [44]	88	High Risk	intra, post	in-hospital (95% < 29 days)
Boyd 1993 [45]	107	High Risk	pre, intra, post, ICU	28 days
Gattinoni 1995 [46]	762	High Risk	ICU	30 days
Lobo 2000 [47]	37	High Risk	intra, post	28 days,
Rivers 2001 [48]	263	Sepsis	ICU	28 days
Chytra 2007 [49]	162	Trauma	ICU	n-hospital (75% <29 days)
Donati 2007 [50]	135	Abdominal	Intra	in-hospital (95% < 30 days)
Kapoor 2008 [51]	27	Cardiac	post	in-hospital (95% < 13 days)
Senagore 2009 [52]	43	Abdominal	Intra	2 days
Jammer 2010 [53]	241	Abdominal	intra	30 days
Jansen 2010 [54]	348	Critically ill	ICU	28 days
Jhanji 2010 [55]	135	Abdominal	post, ICU	in-hospital (75% < 28 days)
Bisgaard 2013 [56]	40	Vascular	Intra, post	30 days
Zheng 2013 [57]	60	Abdominal	Pre, intra, post	in-hospital (75% <27 days)
Peng 2014 [58]	80	Orthopedic	Intra	in-hospital (95% <28 days)
Correa-Gallego 2015 [59]	135	Abdominal	Intra, post	30 days

*Pre* preoperative, *intra* intraoperative, *post* postoperative, *ICU* intensive care unit

**Table 2:** Hemodynamic monitoring used in selected studies

Study	Device	Hemodynamic targets	Urine output threshold	Intervention
Not targeting urine output in either protocol				
Sinclair 1997 [24]	esophageal Doppler	SV		colloids
Polonen 2000 [25]		SvO_2_, Lactate		fluids, dobutamine, vasoactive medication
Rhodes 2002 [26]	PAC	PAWP		fluid boluses, vasoactive agents
Pearse 2005 [27]	LiDCO plus	SV, DO_2_I		colloid, dopexamine
Szakmany 2005 [28]	PiCCO	ITBVI		crystalloid, colloid
Wakeling 2005 [29]	esophageal Doppler	SV		colloids
Forget 2010 [30]	Masimo pulse oximeter	PVI		colloids, vasoactive medication
WenKui 2010 [31]		Lactate		crystalloids, colloids, dopamine, ephedrine
Cecconi 2011 [32]	FloTrac/Vigileo	SV		colloids, vasoactive medication, dobutamine
Challand 2012 [33]	esophageal Doppler	SV		colloid
Bartha 2013 [34]	LiDCO	SV, DO_2_I		fluids, vasoactive medication
Bisgaard 2013 [35]	LiDCO	SVI		colloids, dobutamine, vasoactive medication
Lai 2015 [36]	LiDCO	SVV		Colloids
Targeting urine output only in CFM				
Bishop 1995 [37]	PAC	DO_2_I, VO_2_I, CI	30-50 ml/h	volume, dobutamine
McKendry 2004 [38]	esophageal Doppler	SI	no specific goal mentioned	colloids, blood, vasoactive medication
Benes 2010 [39]	FloTrac/Vigileo	SVV	0.5 ml/kg/h	colloids, dobutamine
Mayer 2010 [40]	FloTrac/Vigileo	CI, SVI	0.5 ml/kg/h	crystalloids, colloids, norepinephrine, dobutamine, vasodilators
McKenny 2013 [41]	esophageal Doppler	SV	0.5 ml/kg/h	colloids
Zakhaleva 2013 [42]	esophageal Doppler	SV, SVR, CO, FTc	0.5-1.0 ml/kg/h	colloids
Osawa 2016 [43]	LIDCO	CI, SVI	0.5 ml/kg/h	crystalloid, dobutamine
Targeting urine ouput in both protocols				
Shoemaker 1988 [44]	PAC	Hct, PvO_2_, PAP, SVR, PWP, PVR, DO_2_, VO_2_	30 mL/h	crystalloids, colloids, vasoactive medication
Boyd 1993 [45]	PAC	DO_2_I	0.5 mL/kg/h	gelatin, dopexamine
Gattinoni 1995 [46]	PAC	CI or SvO_2_	0.5 mL/kg/h	fluids, vasoactive medication
Lobo 2000 [47]	PAC	DO_2_	0.5 mL/kg/h	fluids, dobutamine
Rivers 2001 [48]	computerized spectrophotometer	ScvO_2_, MAP	0.5 mL/kg/h	crystalloid dobutamine, blood transfusions
Chytra 2007 [49]	esophageal Doppler	SV, FTc	1 mL/kg/h	colloids
Donati 2007 [50]		SvO_2_, O_2_ERe	0.5 mL/kg/h	fluids, dobutamine
Kapoor 2008 [51]	FloTrac/Vigileo	CVP, SVV	1 mL/kg/h	colloids, dopamine or other inotropes
Senagore 2009 [52]	esophageal Doppler	SV	0.5 mL/kg/h	colloid
Jammer 2010 [53]		ScvO_2_	0.5 mL/kg/h	crystalloids, colloid
Jansen 2010 [54]	CeVOX	Lactate, ScvO_2_	0.5 mL/kg/h	fluids, vasodilator therapy
Jhanji 2010 [55]	LiDCO	SV	25 mL/h	fluids, dopexamine
Bisgaard 2013 [56]	LiDCO	DO_2_I, SVI	0.5–1.0 mL/kg/h	colloid, dobutamine
Zheng 2013 [57]	FloTrac/Vigileo	CI, SVI, SV	0.5 mL/kg/h	balanced salt solution, colloid, dopamine / norepinephrine, nitroglycerin / ephedrine
Peng 2014 [58]	FloTrac/Vigileo	SVV	0.5 mL/kg/h	Crystalloid, colloid,
Correa-Gallego 2015 [59]	FloTrac/Vigileo	SVV	25 mL/h for 2 consecutive hours	Crystalloid, colloid, albumin bolus infusions

*PAC* pulmonary artery catheter, *PAC+* pulmonary artery catheter with supranormal hemodynamic targets, *pre* preoperative, *intra* intraoperative, *post* postoperative, *ICU* intensive care unit, *ITBVI* intrathoracic blood volume index, *SV* stroke volume, *DO*_*2*_ *I* oxygen delivery index, *PAOP* pulmonary artery occlusion pressure, *CI* cardiac index, *CO* cardiac output, *SVR* systemic vascular resistance, *SVI* systemic vascular index, *PCWP* pulmonary capillary wedge pressure, *DO*_*2*_ oxygen delivery, *PVI* pleth variability index, *GEDI* global end-diastolic volume index, *ELVI* extravascular lung water index, *SvO*_*2*_ mixed venous oxygen saturation, *PAWP* pulmonary artery wedge pressure, *FTc* corrected flow time, *PAWP* pulmonary artery wedge pressure, *SVV* stroke volume variation, *VO*_*2*_ *I* oxygen consumption index, *SI* stroke index, *O*_*2*_ *ERe* oxygen extraction estimate, *ScvO*_*2*_ central venous oxygen saturation, *CVP* central venous pressure, *PvO*_*2*_ venous oxygen pressure, *PAP* pulmonary artery pressure, *PWP* pulmonary wedge pressure, *PVR* pulmonary vascular resistance, *Hct* hematocrit, *VO2* oxygen consumption, *UO* urine output

### Meta-analysis

**Fig. 3:**
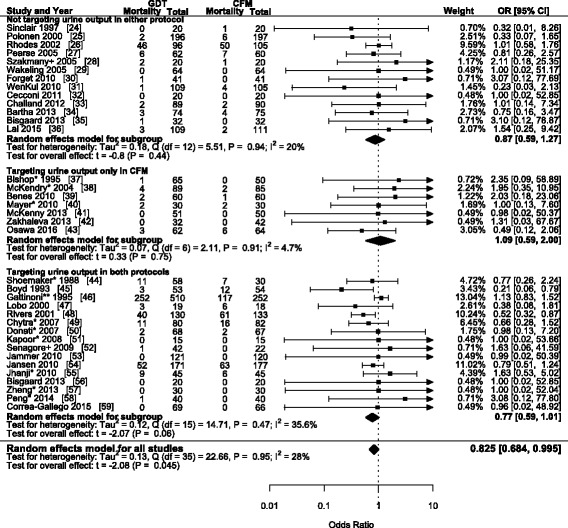
Forest plot of 36 studies reporting 30-day mortality when comparing goal-directed therapy with conventional fluid management. +: mortality follow-up was shorter than 28 days. *: mortality reported as in-hospital mortality. **: mortality data extracted from Kaplan-Meier curve. GDT: goal-directed therapy; CFM: conventional fluid therapy; OR: odds ratio; CI: confidence interval

Because there was no direct data on the effect of targeting urine output on mortality, we first pooled the studies comparing GDT with CFM based on the presence of urine output as a target in either fluid management protocols. Overall, GDT was associated with a decrease in 30-day mortality (OR 0.83; 95% CI 0.68 to 1.00; *P* = 0.04; I^2^ = 28%; *N* = 36) (Fig. 3). However, there was insufficient evidence for a decrease in 30-day mortality due to GDT in all the subgroups. The heterogeneity was low to moderate. The funnel plot is shown in Additional file 2: Figure S1. A slight asymmetry was detected; and identification of eight possible missing studies altered the point estimate (OR 0.75; 95% CI 0.56 to 1.00; *P* = 0.05; I^2^ = 32.8%). The trial sequential analysis is shown in Additional file 3: Figure S2. Despite reaching statistical significance, the required information size of 7400 was not reached and the cumulative Z-score did not cross the monitoring boundaries. This suggests that the results for the beneficial effects of GDT on mortality in this meta-analysis are inconclusive, and the quality of evidence – as assessed by GRADE – is limited.

### Meta-regression analysis

To assess the effects of urine output as a fluid management target from the available data, we performed a meta-regression analysis to estimate a regression line for GDT and CFM with targeting urine output as a secondary variable. There was insufficient evidence to suggest that targeting urine output influences 30-day mortality in a CFM protocol (OR 1.77; 95% CI 0.59-5.34; *P* = 0.305). However, targeting urine output increased 30-day mortality when using GDT (OR 2.66 95% CI 1.06-6.67; *P* = 0.037). After adjusting for study setting, hemodynamic monitoring device, underlying etiology, use of vasoactive medication and year of publication (Table 3), there was insufficient evidence to associate targeting urine output with an effect on 30-day mortality when using a CFM protocol (OR 0.74; 95% CI 0.39-1.38; *P* = 0.334) and a GDT protocol (OR 1.17; 95% CI 0.54-2.56; *P* = 0.685).

**Table 3:** Meta-regression model with 30-day mortality as outcome for conventional and goal-directed fluid therapy

Variable	CFM	GDT
Targeting urine output	0.74 (0.39-1.38)	1.17 (0.54-2.56)
Intensive Care setting (reference)		
Intraoperative setting	0.15 (0.08-0.28)	0.12 (0.05-0.28)
Postoperative setting	0.06 (0.02-0.15)	0.12 (0.03-0.51)
Transpulmonary thermodilution (reference)		
Esophageal Doppler		0.67 (0.21-2.11)
Pulmonary artery catheter		1.27 (0.25-6.35)
Other monitoring devices		0.79 (0.27-2.27)
Other etiologies (reference)		
Abdominal	0.32 (0.15-0.69)	0.76 (0.32-1.77)
High risk	2.13 (0.94-4.81)	2.19 (0.74-6.51)
Inotropic use	1.40 (0.72-2.69)	1.01 (0.40-2.53)
Publication year ^a^	0.97 (0.93-1.02)	1.00 (0.91-1.10)

^a^Publication year was inputted as the years from the mean publication year (2008)

Data reported as odds ratio and 95% confidence interval

### Sensitivity analysis

In the sensitivity analysis excluding studies with a urine output threshold different from the conventional standard of 0.5 ml/kg/h in the targeting urine output group, GDT was associated with a decrease in 30-day mortality (OR 0.78; 95% CI 0.63 to 0.97; *P* = 0.03; I^2^ = 31.8%; *N* = 29, Additional file 4: Figure S3). In the bivariate meta-regression analysis, we found insufficient evidence to suggest that targeting urine output with a threshold of 0.5 ml/kg/h was associated with an increase in 30-day mortality when using a CFM protocol (OR 1.90; 95% CI 0.56 to 6.50; *P* = 0.300) and in a GDT protocol (OR 2.46; 95% CI 0.80 – 7.59; *P* = 0.114). After adjusting for covariates (Table 4), targeting urine output was not associated with a change in 30-day mortality when using a CFM protocol (OR 0.89; 95% CI 0.41 - 1.91; *P* = 0.756) and a GDT protocol (OR 1.08; 95% CI 0.48 – 2.44; *P* = 0.852).

**Table 4:** Meta-regression model of sensitivity analysis with 30-day mortality for conventional and goal-directed fluid therapy

Variable	CFM	GDT
Targeting urine output	0.56 (0.29-1.11)	0.68 (0.34-1.36)
Intensive Care setting (reference)		
Intraoperative setting	0.16 (0.08-0.30)	0.14 (0.08-0.24)
Postoperative setting	0.07 (0.03-0.16)	0.17 (0.05-0.57)
Transpulmonary thermodilution (reference)		
Esophageal Doppler		0.61 (0.23-1.60)
Pulmonary artery catheter		1.80 (0.51-6.33)
Other monitoring devices		0.21 (0.07-0.63)
Other etiologies (reference)		
Abdominal	0.48 (0.21-1.10)	1.14 (0.54-2.38)
High risk	2.36 (0.99-5.67)	1.19 (0.61-2.30)
Inotropic use	1.43 (0.67-3.07)	1.15 (0.47-2.81)
Publication year ^a^	0.95 (0.9-1.00)	0.95 (0.88-1.03)

The sensitivity analysis excluded studies in which the urine output threshold was not 0.5 ml/kg/h

^a^Publication year was inputted as the years from the mean publication year (2008). Data reported as odds ratio and 95% confidence interval

## Discussion

The principal finding of this meta-analysis is that while GDT might decrease 30-day mortality, including urine output as a target may increase 30-day mortality. However, after adjusting for confounders, there is insufficient evidence to associate targeting urine output with an effect on 30-day mortality. Additionally, using the common urine output threshold of 0.5 ml/kg/h, there was insufficient evidence to suggest that targeting urine output affected 30-day mortality. Considering our previous findings that targeting urine output does not prevent acute renal failure [10, 11], our current finding adds further evidence to strongly reconsider the use of urine output as a fluid management target.

Our data shows that GDT is associated with an overall decrease in 30-day mortality, although barely reaching significance. This is partially in agreement with previously published meta-analyses on GDT and mortality. While one meta-analysis in surgical patients reported that GDT was associated with a decrease in mortality [66], another meta-analysis in surgical patients found no such effect [67]. The difference in mortality between these two meta-analyses may be due to studies published after the publication of the meta-analysis by Brienza et al. [66]. The disagreement between the meta-analysis by Corcoran et al. [67] and our meta-analysis may be due to three reasons: the inclusion of newer studies, the addition of critical care studies, and the follow-up period for mortality. Additionally, the meta-analysis by Zhang et al. showed that patients with severe sepsis or septic shock receiving GDT had a similar risk of mortality compared with those in the control group [68]. Nevertheless, as the optimal information size metric suggests, the currently available pool of studies may be insufficient to conclusively state any effect of GDT on mortality.

This meta-analysis supports the hypothesis that oliguria is likely an epiphenomenon rather than a modifiable risk factor. In a perioperative setting, low urine output is common in the first 24 h after surgery and in the absence of other issues it does not reliably reflect fluid status [5]. Moreover, urine output is influenced by factors other than the hemodynamic status [69, 70]. Surgical trauma and physical stress in critical illness cause the release of neuro-hormonal factors which influence glomerular filtration pressure or water reabsorption in the collecting duct, such as catecholamines, arginine vasopressin and the renin-angiotensin-aldosterone system. While these neuro-hormonal factors are also upregulated in hypovolemia resulting in oliguria, the perioperative or critical care setting itself does promote the occurrence of oliguria. Additionally, anesthetic techniques and medication can affect neuro-hormonal factors as well as vasomotor tone. Moreover, using urine output to guide fluid management is inherently flawed due to the delayed response. Evaluating the urinary response to a fluid challenge is generally possible after at least 15-30 min, and is limited by the lack of a clear dose-response relationship. In contrast, hemodynamic parameters such as cardiac output are dynamic variables which are influenced within a short interval after a fluid challenge is given and for most variables a dose-response relationship has been given. Thus, the primary cause of oliguria may not be affected by fluid administration, or may already have been resolved by acting on another target.

In light of this, the use of permissive oliguria has already been advocated in ERAS protocols, primarily to avoid excess fluid loading [7]. In patients managed by hemodynamic targets with a better correlation to fluid status, the occurrence of oliguria due to hemodynamic causes is unlikely, which favors the exclusion of urine output as a target for fluid resuscitation. Considering this, the current paradigm that urine output reflects renal injury and – perhaps indirectly – increases mortality needs to be revisited [12, 14]. In most – if not all – cases, oliguria is most likely an epiphenomenon of an underlying problem. A recent study showed that after adjusting for confounders while intraoperative urine output was not associated with postoperative morbidity, total intraoperative fluid intake and postoperative fluid boluses for hypotension and low urine output were associated with an increase in postoperative morbidity [59]. This strongly suggests that urine output should not be a target in a fluid management protocol to improve outcome.

This meta-analysis has several important limitations. The main limitation is the various sources of heterogeneity. The I^2^ statistic showed low to moderate heterogeneity in most analyses. However, considering the different hemodynamic targets, fluid types, vasopressor use, monitoring devices, underlying etiologies, clinical settings and mortality follow-up used in these studies, assuming that the heterogeneity is as low as suggested by the I^2^ statistic would be imprudent. Because some studies did not report data of fluids infused as a statistical measure and data regarding the amount of urine output were rarely reported at all, further analysis of these data points was not possible. Despite the use of a random-effects model and a bi- and multivariate approach to a meta-regression analysis, the effects of between-trial differences are most likely not completely taken into account [19, 20]. Since our findings are based on between-trial statistical analyses, given the large differences between the included studies, the interpretation of these findings – even after adjusting for operative setting, underlying etiology and other confounders - should be done with care. Understandably, given the absence of trials primarily investigating the effects of urine output as a target, to account for all the possible sources of heterogeneity within the currently available literature would be impractical and the inability to do so is currently an inevitable limitation. However, after acknowledging this limitation, our findings are currently the only assessment of the effects of targeting urine output on mortality, and are supported by the observations from various trials [6, 59].

Another important limitation is the low number of studies given the available literature on GDT. The potential for robust conclusions by using meta-regression is limited by the number of studies [71]. However, to ensure that heterogeneity was limited as much as possible, several of the larger - and perhaps more convincing – trials were excluded. The three recent large studies - ARISE, PROCESS and PROMISE - were not included in this meta-analysis, due to meeting our exclusion criterion of vague CFM protocols [72–74]. While their exclusion may limit the generalization of our findings, the strict inclusion and exclusion criteria removes bias caused by some of the heterogeneity. Given that our main objective was to assess the effect of targeting urine output on 30-day mortality, removing as many sources of heterogeneity as possible strengthens our findings. Similarly, despite the absence of these large trials, the mortality rate in most studies is close to the estimated 30-day mortality rate in elective – high risk – surgery (~7%) and critical care (~15%) [75–77]. Additionally, a slight asymmetry was found in the funnel plot, and after applying the ‘trim and fill’ method, the effect size of eight possible missing studies were added to the analysis. In combination with the trail sequential analysis, this suggests insufficient evidence to support a difference in 30-day mortality between GDT and CFM, despite the analysis in Fig. 3, and illustrates the dependence on adequate sample size to establish definite conclusions.

## Conclusion

In conclusion, based on the currently available literature, we found that while GDT might decrease 30-day mortality, including urine output as a target may increase 30-day mortality. However, the principal finding of this meta-analysis is that after adjusting for confounders, there is insufficient evidence to associate targeting urine output with an effect on 30-day mortality. This suggests that oliguria is not a modifiable risk factor for mortality, and using diuresis to guide fluid management may not affect survival. However, the paucity of direct data illustrates the need for further research on whether oliguria is just an epiphenomenon and whether ‘permissive oliguria’ should be a key component of fluid management protocols.

## Additional files

Additional file 1: Tables with the search strategy used in the MEDLINE and EMBASE databases. Tables reporting the amount of fluids infused, urine output data, and the characteristics of observational studies. (PDF 184 kb)
Additional file 2:
**Figure S1.** Funnel plot used to assess the presence of publication bias in the performed analysis. (PDF 53 kb)
Additional file 3:
**Figure S2.** Trial sequential analysis for cumulative meta-analysis. Data is analyzed cumulatively in order of year of publication, and the optimal information size (sample size) is 7400 patients to find a 25% relative risk reduction with a power of 80% and an alpha of 0.05. (PDF 6 kb)
Additional file 4:
**Figure S3.** Forest plot of sensitivity analysis (urine output threshold 0.5 ml/kg/h) on 30-day mortality when comparing goal-directed therapy with conventional fluid management. +: mortality follow-up was shorter than 28 days. *: mortality reported as in-hospital mortality. **: mortality data extracted from Kaplan-Meier curve. GDT: goal-directed therapy; CFM: conventional fluid therapy; OR: odds ratio; CI: confidence interval. (PDF 51 kb)

## Abbreviations

CFM: Conventional fluid management
ERAS: Early recovery after surgery
GDT: Goal-directed therapy
OR: Odds ratio
