# Supplementary material for: Targeting urine output and 30-day mortality in goal-directed therapy: a systematic review with meta-analysis and meta-regression
Source: BMC Anesthesiol. 2017 Feb 10;17:22. doi: 10.1186/s12871-017-0316-4 (PMC5303289; doi:10.1186/s12871-017-0316-4)
Supplement: Additional file 1: — Tables with the search strategy used in the MEDLINE and EMBASE databases. Tables reporting the amount of fluids infused, urine output data, and the characteristics of observational studies. (PDF 184 kb) [file 12871_2017_316_MOESM1_ESM.pdf]

Table S1 Search strategy in MEDLINE database through PubMed

#49	((#41 AND #48))	446
#48	((#44 OR #47))	
#47	((#45 OR #46))	
#46	"controlled trial"	
#45	trial	
#44	((#42 OR #43))	
#43	"randomised"	
#42	"randomized"	
#41	((#31 AND #40))	
#40	((#12 AND #39))	
#39	((#32 OR #33 OR #34 OR #35 OR #36 OR #37 OR #38))	
#38	fluid	
#37	"fluid resuscitation"	
#36	"fluid loading"	
#35	"fluid administration"	
#34	"fluid management"	
#33	"fluid therapy"	
#32	"fluid therapy"[MeSH]	
#31	((#13 OR #14 OR #15 OR #16 OR #17 OR #18 OR #19 OR #20 OR #21 OR #22 OR #23 OR #24 OR #25 OR #26 OR #27 OR #28 OR #29 OR #30))	
#30	outcome	
#29	"urinary output"	
#28	"urine production"	
#27	"urine output"	
#26	"diuresis"	
#25	"mortality rate"	
#24	"loss of life"	
#23	decease	
#22	lethality	
#21	fatality	
#20	survival	
#19	death	
#18	mortality	
#17	"mortality"[MeSH]	
#16	"acute kidney injury"	
#15	"complications"	
#14	"organ dysfunction"	
#13	"organ failure"	
#12	(#1 OR #2 OR #3 # OR #4 OR #5 OR #6 OR #7 OR #8 OR #9 OR #10 OR #11)	
#11	"supranormal"	
#10	"goal directed therapy"	
#9	"goal-directed therapy"	
#8	optimisation	
#7	optimization	
#6	"cardiac index"	
#5	"stroke volume"	
#4	"cardiac output"	

#3	"hemodynamic target"	
#2	"goal-directed"	
#1	"goal directed"	

Table S2 Search strategy in EMBASE database

#47	#39 AND #46	890
#46	#42 OR #45	
#45	#43 OR #44	
#44	"controlled trial"	
#43	trial	
#42	#40 OR #41	
#41	"randomised"	
#40	"randomized"	
#39	#30 AND #38	
#38	#12 AND #37	
#37	((#31 OR #32 OR #33 OR #34 OR #35 OR #36))	
#36	fluid	
#35	"fluid resuscitation"	
#34	"fluid loading"	
#33	"fluid administration"	
#32	"fluid management"	
#31	"fluid therapy"	
#30	((#13 OR #14 OR #15 OR #16 OR #17 OR #18 OR #19 OR #20 OR #21 OR #22 OR #23 OR #24 OR #25 OR #26 OR #27 OR #28 OR #29))	
#29	outcome	
#28	"urinary output"	
#27	"urine production"	
#26	"urine output"	
#25	"diuresis"	
#24	"mortality rate"	
#23	"loss of life"	
#22	decease	
#21	lethality	
#20	fatality	
#19	survival	
#18	death	
#17	mortality	
#16	"acute kidney injury"	
#15	"complications"	
#14	"organ dysfunction"	
#13	"organ failure"	
#12	(#1 OR #2 OR #3 OR #4 OR #5 OR #6 OR #7 OR #8 OR #9 OR #10 OR #11)	
#11	"supranormal"	
#10	'goal-directed'	
#9	'goal directed'	
#8	optimisation	
#7	optimization	
#6	"cardiac index"	
#5	"stroke volume"	
#4	"cardiac output"	
#3	"hemodynamic target"	
#2	'goal-directed therapy'	

#1	'goal directed therapy'	

Supplemental Digital Content Table S3 Amount of fluids infused during the relevant study period.

Study	Total fluid amount GDT (mL)	Total fluid amount CFM (mL)
**Not targeting urine output in either protocol**		
Sinclair 1997	1475	1000
Polonen 2000	3193	2772
Rhodes 2002	4953	4295
Pearse 2005	2962	2164
Szakmany 2005	5458	5298
Wakeling 2005	5000	4500
Forget 2010	5777	6835
WenKui 2010	2800	2800
Cecconi 2011	6229	3293
Challand 2012	5309	4010
Bartha 2013	1310	1197
Bisgaard 2013	7236	8953
Lai 2015	5369	4532
**Targeting urine output only in CFM**		
Bishop 1995	5496	6165
McKendry 2004	2020	1370
Benes 2010	3746	3729
Mayer 2010	4528	4494
McKenny 2013	2000	2500
Zakhaleva 2013	5300	5600
Osawa 2016	1056	894
**Targeting urine output in both protocols**		
Shoemaker 1988	-	-
Boyd 1993	5075	4845
Gattinoni 1995	-	-
Lobo 2000	7200	6600
Rivers 2001	4981	3499
Chytra 2007	4516	3599
Donati 2007	4391	4285
Kapoor 2008	-	-
Senagore 2009	3400	3000
Jammer 2010	3875	6490
Jansen 2010	3019	2390
Jhanji 2010	1879	1743
Bisgaard 2013	4314	3616
Zheng 2013	2650	3950
Peng 2014	2100	2600
Correa-Gallego 2015	2000	2900

Pre: preoperative; intra: intraoperative; post: postoperative; ICU: intensive care unit.

Supplemental Digital Content Table S4 Available urine output data from the selected studies

	Intraoperative urine output			Postoperative urine output		
**Study**	**GDT**	**CFM**	**P**	**GDT**	**CFM**	**P**
**Not targeting urine output**						
Szakmany 2005	757 mL ± 533	755 mL ± 528	NS			
Cecconi 2011	1225 mL (IQR 650 - 1375)	300 mL (IQR 100 - 475)	<0.0001			
Challand 2012	655 mL ± 302	388 mL ± 355	<0.001			
Bartha 2013	400 mL (range 0 - 1900)	300 mL (range 0 - 1300)	NS	400 mL (range 0 - 2275)	350 mL (range 25 - 4800)	NS
**Targeting urine output**						
Gattinoni 1995				95.8 mL ± 50.1	CIG: 102 mL ± 49.5<br>O_2G: 95.5 mL ± 49.5	0.274
Kapoor 2008				230* mL	140* mL	NS
Jammer 2010	1.1 ml/kg/h ± 1.5	1.5 ml/kg/h ± 1.5	0.020	83 mL 1 ± 445	1104 mL ± 449	<0.001
Zheng 2013	618 mL ± 239	800 mL ± 304	<0.001	518 mL ± 330	870 mL ± 304	<0.001
Peng 2014	1.98 ml/kg/h (IQR 1.29 - 2.63)	2.20 ml/kg/h (IQR 1.53 - 3.25)	NS			
Correa-Gallego 2015	200 mL ± 100	300 mL ± 200	0.10	900 mL ± 600	1000 mL ± 500	0.07

Data as reported by the respective studies as either mean ± standard deviation or median (interquartile range (IQR) or range), in mL or mL/kg/h. *: approximated from figure. GDT: goal-directed therapy; CFM: conventional fluid management; CIG: cardiac index guided group; O_2G: oxygen saturation guided group. NS: not statistically significant.

Supplemental Digital Content Table S5 Characteristics of observational studies included

Study	Total number	Type of patient	Timing	Mortality (GDT vs CFM), follow up
**Not targeting urine output in either protocol**				
Hussien 2011	25	Abdominal	intra	1 vs 0, 10 days
See 2014	612	Critically ill	ICU	90 vs 148, 30 days
Thomson 2014	264	Cardiac	Post	0 vs 2 ,30 days
Cannesson 2015	330	Abdominal, pelvic	intra	2 vs 1, 30 days
**Targeting urine output in both protocols**				
Sivayoham 2012	174	Critically ill	ICU	22 vs 33, 30 days
Reydellet 2013	50	Abdominal	Intra, post	1 vs 4, 30 days

Pre: preoperative; intra: intraoperative; post: postoperative; ICU: intensive care unit.

Supplemental Digital Content Table S6 Hemodynamic monitoring used in observational studies

Study	Device	Hemodynamic targets	Urine output threshold	Intervention
**Not targeting urine output in either protocol**				
Hussien 2011	Oesophageal Doppler	SV		colloids
See 2014		PP, SV		crystalloids
Thomson 2014	LiDCOplus	SV		colloids, cystalloids, blood products
Cannesson 2015	EV 1000, Edwards	SV, SVV, CI		crystalloids
**Targeting urine output in both protocols**				
Sivayoham 2012		$SVO_{2,}$ CI,	0.5 ml/kg/h	crystalloids, colloids, vasoactive medication
Reydellet 2013	FloTrac-Vigileo	MAP, CO, CI, SV, SVV, ScvO2	no specific goal mentioned	colloids, crystalloids vasoactive medication

SV: stroke volume; PP: pulse pressure; SVV: stroke volume variation; CI: cardiac index; SvO_2: mixed venous oxygen saturation; MAP: mean arterial pressure; CO: cardiac output; $ScvO_2$: central venous oxygen saturation;
