# Supplementary figures and images for: Targeting urine output and 30-day mortality in goal-directed therapy: a systematic review with meta-analysis and meta-regression
Source: BMC Anesthesiol. 2017 Feb 10;17:22. doi: 10.1186/s12871-017-0316-4 (PMC5303289; doi:10.1186/s12871-017-0316-4)

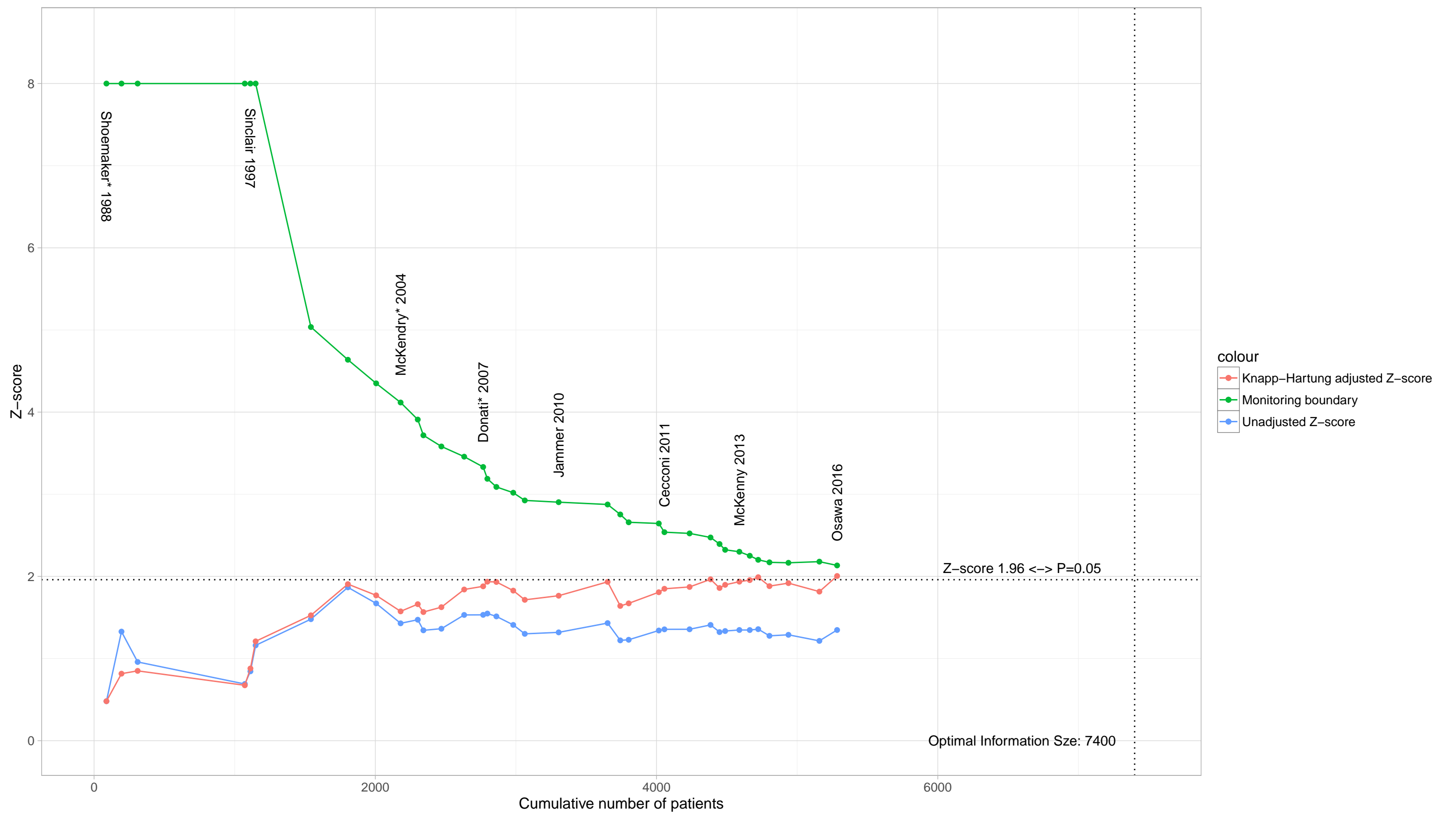

Supplement: Additional file 3: — Figure S2. Trial sequential analysis for cumulative meta-analysis. Data is analyzed cumulatively in order of year of publication, and the optimal information size (sample size) is 7400 patients to find a 25% relative risk reduction with a power of 80% and an alpha of 0.05. (PDF 6 kb) [file 12871_2017_316_MOESM3_ESM.pdf]

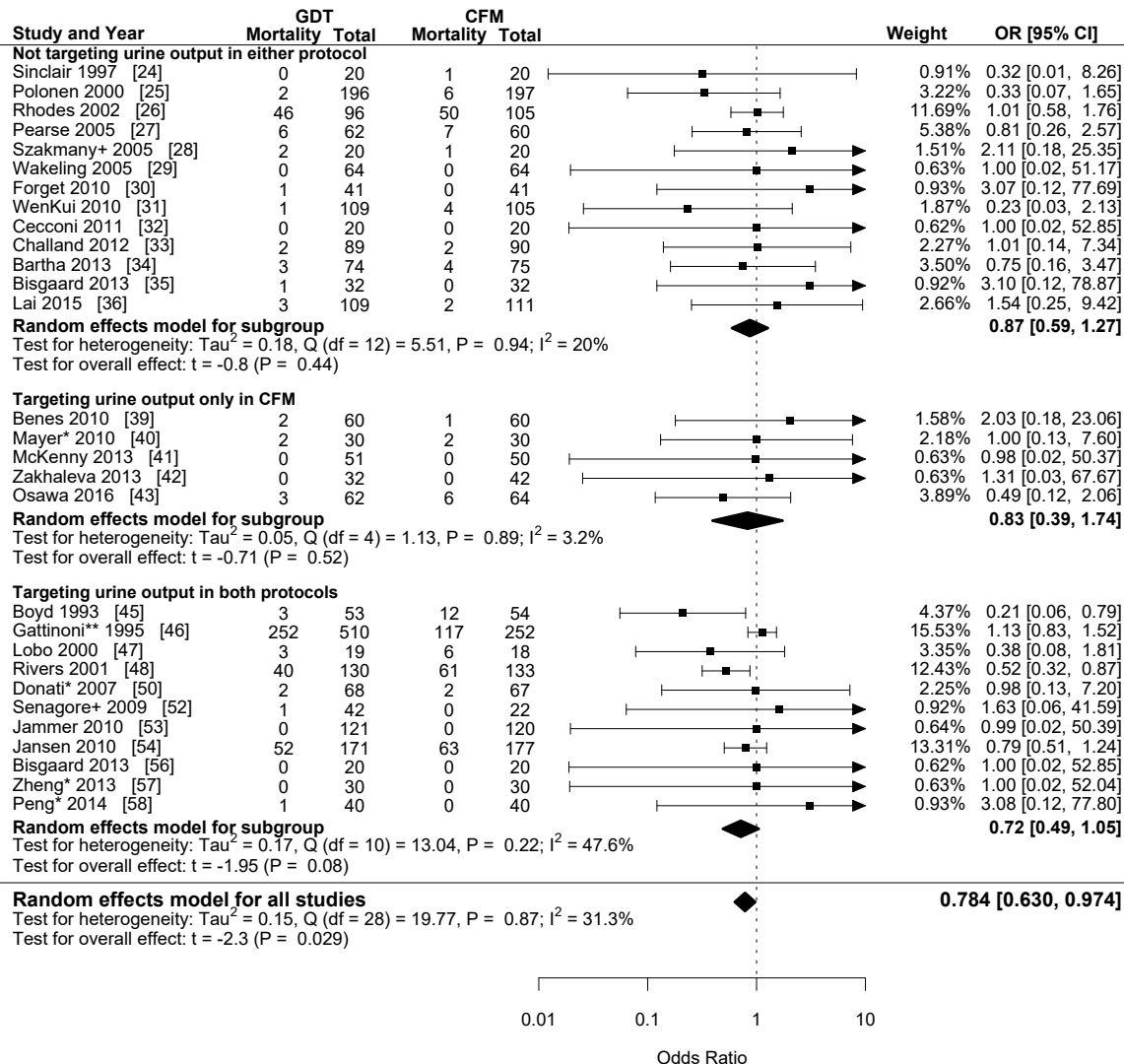

Supplement: Additional file 4: — Figure S3. Forest plot of sensitivity analysis (urine output threshold 0.5 ml/kg/h) on 30-day mortality when comparing goal-directed therapy with conventional fluid management. +: mortality follow-up was shorter than 28 days. *: mortality reported as in-hospital mortality. **: mortality data extracted from Kaplan-Meier curve. GDT: goal-directed therapy; CFM: conventional fluid therapy; OR: odds ratio; CI: confidence interval. (PDF 51 kb) [file 12871_2017_316_MOESM4_ESM.pdf]
