# Supplementary material for: Targeting urine output and 30-day mortality in goal-directed therapy: a systematic review with meta-analysis and meta-regression
Source: BMC Anesthesiol. 2017 Feb 10;17:22. doi: 10.1186/s12871-017-0316-4 (PMC5303289; doi:10.1186/s12871-017-0316-4)
Supplement: Additional file 2: — Figure S1. Funnel plot used to assess the presence of publication bias in the performed analysis. (PDF 53 kb) [file 12871_2017_316_MOESM2_ESM.pdf]

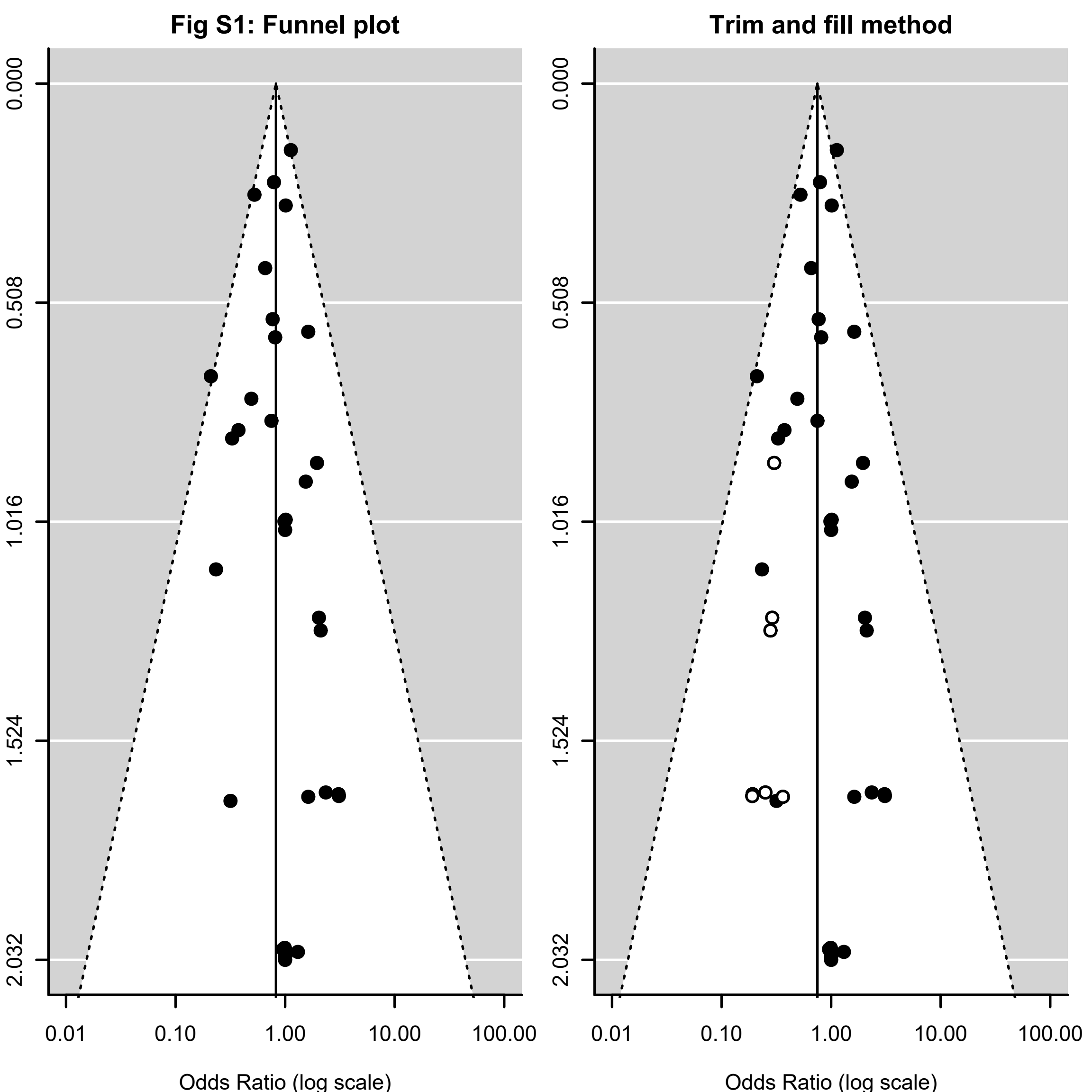

Fig S1: Funnel plot
Trim and fill method
0.000
0.508
1.016
1.524
2.032
0.01
0.10
1.00
10.00
100.00
Odds Ratio (log scale)
